# Supplementary material for: Construction of an lncRNA-mediated ceRNA network to investigate the inflammatory regulatory mechanisms of ischemic stroke
Source: PLoS One. 2025 Jan 23;20(1):e0317710. doi: 10.1371/journal.pone.0317710 (PMC11756804; doi:10.1371/journal.pone.0317710)
Supplement: S1 Fig — From left to right, they are intergenic lncRNA (red); intronic lncRNA (green); antisense lncRNA (blue); and sense lncRNA (purple). S1B: Circular diagram of lncRNA distribution on chromosomes. The outermost layer is the chromosome ring of the genome, and from outside to inside are sense lncRNA (green), intergenic region lncRNA (red), intronic lncRNA (blue), and antisense lncRNA (gray). (DOCX) [file pone.0317710.s001.docx]

S1A


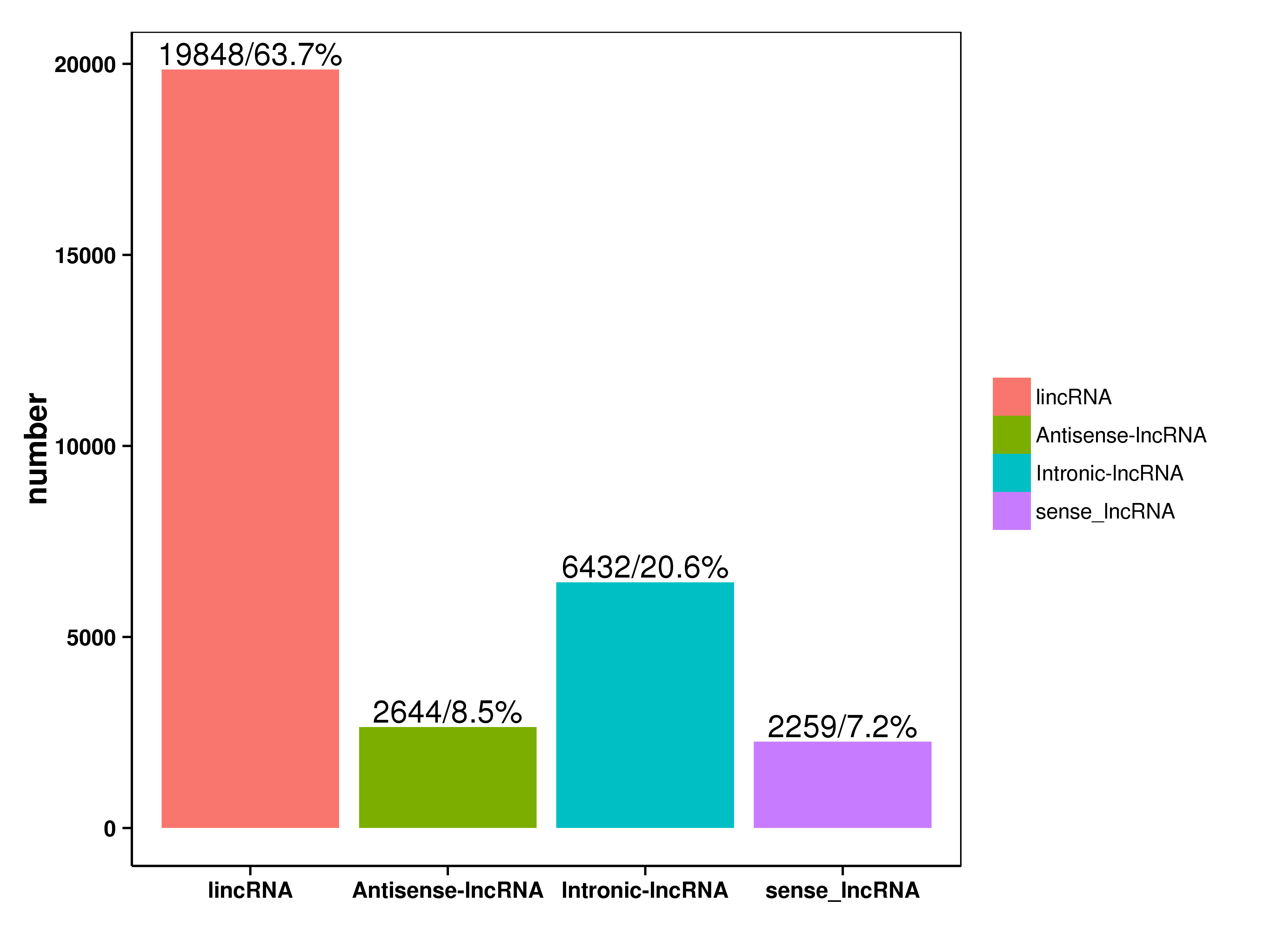


S1B


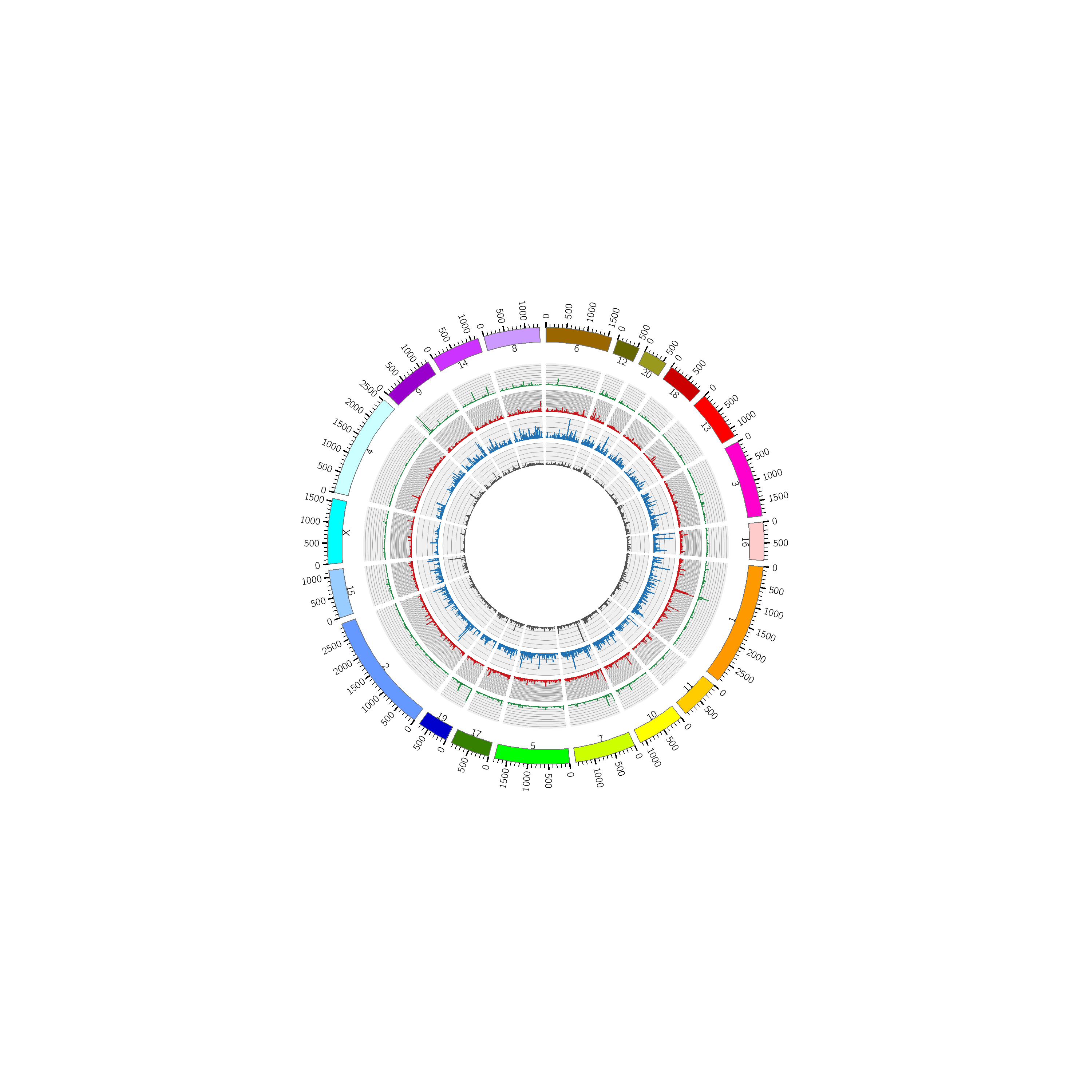


S1A;Classification map of 31183 lncRNAs obtained by sequencing. From left to right, they are intergenic lncRNA (red); intronic lncRNA (green); antisense lncRNA (blue); and sense lncRNA (purple).

S1B:Circular diagram of lncRNA distribution on chromosomes. The outermost layer is the chromosome ring of the genome, and from outside to inside are sense lncRNA (green), intergenic region lncRNA (red), intronic lncRNA (blue), and antisense lncRNA (gray).
