# Supplementary material for: Construction of an lncRNA-mediated ceRNA network to investigate the inflammatory regulatory mechanisms of ischemic stroke
Source: PLoS One. 2025 Jan 23;20(1):e0317710. doi: 10.1371/journal.pone.0317710 (PMC11756804; doi:10.1371/journal.pone.0317710)
Supplement: S1 Table — (DOCX) [file pone.0317710.s002.docx]

| Primer name | Primer sequence forward (5′-3′)  Table1 Primer sequence | Primer sequence reverse (5′-3′) |
| --- | --- | --- |
| MSTRG.151823.1 | CCCCAAACTGAAACTCTGTCC | GGTAAACGAGGGGTGTAGATG |
|  | AACTGAAACTCTGTCCGCACC | ACTTACCACTCCCCAAAACCC |
| MSTRG.14764.1 | GTAAGGTCAAGCAGATTCAGG | GTCCCAGCCCATTTTTTTTCT |
|  | GAACTACACCCTCAACCCAAA | ATGTTCAGTCGGGAGTAGATT |
| MSTRG.82425.1 | CCCTGTCCAAGATGTAATGTT | CCCCCTTTCTTCTTTTTCCTG |
|  | ATGATGCTGATGATGGAACCT | GACAGGTTTCTTTGCTTTGAC |
| MSTRG.29312.2 | ACAGAAGAGGCAGGAGGGCAA | GTCATCATCGGCGTGTCAGTC |
|  | ACATTTACAGACAAGAGCCAG | GTAGAGATGAGGCTTGGAAGT |
| MSTRG.92442.2 | AGCAGCAACTTCTCTTCAACA | CCTCCAAACACCAAGACTATC |
|  | ATCAGTTCCTTTCCCCCCATC | ACAATCACCTTCTCTTCTTCC |
| MSTRG.24573.12 | ACATCACACACACACACACAC | AGAACCCCAACCAACCAACAC |
|  | CACACACACCTCACTCAACAC | TGTGAGAGGTGTGTGTGTGAG |
| MSTRG.179535.4 | CTTACTGTGGGTTTACCGTTT | GAAACCTGAAGAAATCCAACC |
|  | GGTGATGAGGTTTCTGGGCTG | AATGGAGGCTGCTGGAACCGA |
| MSTRG.112172.24 | TGAAGGAAGGTTGGGAGAAGT | GGCATAGTGAGCAGAAAGGAA |
|  | CTTTGTGCTTCTGTGTTGACT | CAAGAGGCAGAACAGCACAAA |
| MSTRG.82516.1 | CCCTTTATCTAACCATCTCAC | AGGGTAGTGGTTTCAAGCAGT |
|  | CATCTCACAAGGCTTCAACCA | GTAAAGATGGTTGTGGCAGGT |
| MSTRG.272494.1 | AGGTAAGCATCTGGCAGTAGG | CTTTGGGTGTGAATGCTATGG |
|  | AAACCGCTGGAACTTGACTGA | GAATGCTATGGTATGTGTGGA |
| MSTRG.68448.1 | AAAAGTAACGCAACGCCAGGT | CTTCCTAACGCACACTACACA |
|  | GAAAGGTGGAGGCAGGAGGAT | ATCACAGAGCACTCCCGTCCT |
| MSTRG.189479.6 | AGGTTTAGCAGATCAGGAGTT | TTCTCCATCCCTTTCCTTATC |
|  | CAAGGGAGTACCATGAACCAG | GGCTGATTATGTACTTGTGGG |
| MSTRG.191888.1 | GGGAGATGTAGGTATGGCTTG | AGTGGCTGGGAGGGCTATCAA |
|  | CCATAGTCATTCTTCCGATTC | ACAGACGATTTTAGTGGCTTC |
| MSTRG.95251.1 | CCACAAGTTAGAAGCAGAAAG | TCTTACAACACCCACACTCTC |
|  | GAAGCAGAAAGAAAGAGACAG | GCAATAGATAAGATGGCTCCT |
| MSTRG.191887.1 | TGAGGCTTGGTACTTCTTCTT | TGGTAATGTTTTGGTGGTCCT |
|  | CAGAACTCATCCCACCTACAG | ACCTCAAATGCTATGCTGGAC |
| MSTRG.245764.1 | GAATCCATCCTACCTCTCTCT | TGACTGAAGAGAACTGAAGGG |
|  | CCTTCCGCATTGTCTCCCTTG | CCCAGGCGATTCCACCATTAG |
| MSTRG.114383.1 | AGGAAGAGGGTGAGGAAGAGG | CTCATCTTCCCCTTTTCAGCC |
|  | GCAAGGTGGATTAGCAAGTGA | TTCTATCTGACTTGCCTGACT |
| Rat-β-actin | CACCCGCGAGTACAACCTTC | CCCATACCCACCATCACACC |
| Furin | AGCAGCAGGTAGCCAAGCAAA | CCTGTTGTCATTCATCTGCGT |
| Tacc3 | ATCCTCTGTTCTTCGCCTGTC | TCTCTCTGCCAGTTCTCCTTG |
| Ptgs2 | TACGCCTGAGTTTCTGACAAG | TTGTAAGTTGGTGGGCTGTCA |
| Lgals3 | TGACAGTGCCCTACGATATGC | TTGAAGAAGTGGGGCATGAAG |
| Serpine1 | ATGTGGTCTTCTCTCCCTACG | TGGCTGAGTCTGTGTCCTTGT |
| Myh9 | CTTCATCAATAACCCGCTGGC | TCATCCTTGTTCACCTTCACC |
| Bag3 | GATGGCGTCAGGAAGGTTCAG | TCTGCTGTGCTGCTGGGGTTT |
| Flna | TTGATACACGGGACGCTGAGA | TCGTTGGTTGTGCTTGCGGTG |
| Jak3 | TTCTCTTTTGGGGACTACTTG | TCAAGGATGGCACTGGTCAAA |
| Mcm2 | TGGAGGAAGAAGAGGATGGAG | TCATCTTCTTCGTCGCTGTCA |
| IL6R | TGGCAAATGGTACGGTGACGA | AATAGTGCCCAGTGTCGTTGA |
| miR-351-5p RT | GTCGTATCCAGTGCAGGGTCCGAGGTATTCGCACTGGATACGACAGGGAC | |
| miR-351-5p-F | TCCCCTGAGGAGCCCTTTG | |
| miR-351-5p-R | GTGCAGGGTCCGAGGT | |
| miR-370-3p RT | GTCGTATCCAGTGCAGGGTCCGAGGTATTCGCACTGGATACGACACCAGGT | |
| miR-370-3p-F | TGCCTGCTGGGGTGGA | |
| miR-370-3p-R | GTGCAGGGTCCGAGGT | |
| miR-139-3p RT | GTCGTATCCAGTGCAGGGTCCGAGGTATTCGCACTGGATACGACCTCCAAC | |
| miR-139-3p-F | TGGAGACGCGGCCCT | |
| miR-139-3p-R | GTGCAGGGTCCGAGGT | |
| miR-674-5p RT | GTCGTATCCAGTGCAGGGTCCGAGGTATTCGCACTGGATACGACGTGGTAC | |
| miR-674-5p -F | GCACTGAGAGTGTG | |
| miR-674-5p -R | GTGCAGGGTCCGAGGT | |
| U6 RT | CTCAACTGGTGTCGTGGAGTCGGCAATTCAGTTGAGAAAAATATGG | |
| U6-F | CTCGCTTCGGCAGCACA | |
| U6-R | AACGCTTCACGAATTTGCGT | |
