# Supplementary material for: Construction of an lncRNA-mediated ceRNA network to investigate the inflammatory regulatory mechanisms of ischemic stroke
Source: PLoS One. 2025 Jan 23;20(1):e0317710. doi: 10.1371/journal.pone.0317710 (PMC11756804; doi:10.1371/journal.pone.0317710)
Supplement: S2 Table — (DOCX) [file pone.0317710.s003.docx]

Table S2：Summary of the mapping data from the cortex tissue

| Sample-ID | Clean Reads | Mapped Reads | Uniq Mapped Reads | Multiple Mapped Reads | Q20(%) | Q30(%) |
| --- | --- | --- | --- | --- | --- | --- |
| T01 | 69,323,324 | 66,645,539(96.14%) | 61,402,683(88.57%) | 5,242,856(7.56%) | 97.89 | 93.97 |
| T02 | 66,735,994 | 64,372,321(96.46%) | 59,540,821(89.22%) | 4,831,500(7.24%) | 97.97 | 94.1 |
| T03 | 71,072,850 | 68,056,514(95.76%) | 62,719,202(88.25%) | 5,337,312(7.51%) | 97.79 | 93.75 |
| T04 | 77,197,490 | 74,004,299(95.86%) | 67,442,367(87.36%) | 6,561,932(8.50%) | 97.97 | 94.16 |
| T05 | 69,314,306 | 66,414,860(95.82%) | 60,571,267(87.39%) | 5,843,593(8.43%) | 97.9 | 93.99 |
| T06 | 67,918,358 | 65,047,202(95.77%) | 60,214,975(88.66%) | 4,832,227(7.11%) | 97.99 | 94.26 |
| T07 | 72,494,446 | 69,081,816(95.29%) | 63,985,013(88.26%) | 5,096,803(7.03%) | 98.02 | 94.25 |
| T08 | 75,756,802 | 72,724,724(96.00%) | 66,997,068(88.44%) | 5,727,656(7.56%) | 97.95 | 94.11 |
| T09 | 71,646,638 | 68,859,965(96.11%) | 63,911,627(89.20%) | 4,948,338(6.91%) | 98.13 | 94.48 |
| T10 | 85,438,172 | 81,964,424(95.93%) | 75,735,741(88.64%) | 6,228,683(7.29%) | 98 | 94.21 |
