# Supplementary material for: Construction of an lncRNA-mediated ceRNA network to investigate the inflammatory regulatory mechanisms of ischemic stroke
Source: PLoS One. 2025 Jan 23;20(1):e0317710. doi: 10.1371/journal.pone.0317710 (PMC11756804; doi:10.1371/journal.pone.0317710)
Supplement: S8 Table — (DOCX) [file pone.0317710.s009.docx]

Table S7 miRNA Data Evaluation Statistics

| **Samples** | **BMK‑ID** | **Raw reads** | **Low quality reads** | **Containing'N'reads** | **Length <18** | **Length >30** | **Clean reads** | **Q30(%)** |
| --- | --- | --- | --- | --- | --- | --- | --- | --- |
| 1-3 C | S01 | 17512893 | 0.00% | 0.00% | 9.77% | 8.30% | 14348469 | 96.43 |
| C 4 | S02 | 16208465 | 0.00% | 0.00% | 18.77% | 3.87% | 12538474 | 96.5 |
| C 6 | S03 | 16818698 | 0.00% | 0.00% | 24.18% | 4.23% | 12041706 | 96.78 |
| C 7 | S04 | 21874946 | 0.00% | 0.00% | 15.03% | 6.60% | 17143329 | 96.6 |
| C 9 | S05 | 20678461 | 0.00% | 0.00% | 8.83% | 14.77% | 15814234 | 96.81 |
| 假 1 C | S06 | 13946023 | 0.00% | 0.00% | 7.43% | 3.10% | 12478701 | 95.89 |
| 假 2C | S07 | 24997121 | 0.00% | 0.00% | 22.19% | 1.93% | 16582131 | 98.6 |
| 假2 C | S08 | 14707225 | 0.00% | 0.00% | 6.12% | 2.64% | 13417704 | 96.31 |
| 假4 C | S09 | 14613842 | 0.00% | 0.00% | 5.51% | 3.96% | 13228750 | 96.58 |
| 假5 C | S10 | 12376342 | 0.00% | 0.00% | 8.60% | 3.21% | 10913605 | 96.39 |
