# Supplementary material for: Construction of an lncRNA-mediated ceRNA network to investigate the inflammatory regulatory mechanisms of ischemic stroke
Source: PLoS One. 2025 Jan 23;20(1):e0317710. doi: 10.1371/journal.pone.0317710 (PMC11756804; doi:10.1371/journal.pone.0317710)
Supplement: Attachment — Submitted filename: Response to Reviewers.docx [file pone.0317710.s021.docx]

Dear Editors,

We thank you very much for giving us the opportunity to revise our manuscript. We greatly appreciate you and the reviewers for your positive and constructive comments and suggestions on our manuscript entitled “Construction of an lncRNA-mediated ceRNA network to investigate the inflammatory regulatory mechanisms of ischemic stroke.” Professional comments from the reviewers have been very helpful in improving the quality of this article. As required, we have revised the manuscript. Furthermore, we have also carefully checked the text and figures in this proof. The main corrections in the paper and the responses to the reviewers’ comments are as follows:

**Reviewer #1:**

Major Comments:

1. **Comment:**The authors conducted a deep-sequencing approach for mRNA, miRNA, and lncRNA using a rat model for ischemic stroke. I have the impression that this study lacks experiments to confirm the regulatory interactions predicted by their bioinformatics approach. It could be done in certain cell lines by knocking down identified miRNA or lncRNA. I cannot be positive about the publication of this study without validation data.

**Response**: Thank you for your valuable feedback on our research. We take your suggestions regarding the validation of our regulatory network predictions very seriously. Below, we provide further clarification on the design and theoretical foundations of our study to demonstrate the reliability and scientific basis of our findings.

1. Correlation between High-Throughput Sequencing and qRT-PCR Validation of Expression Patterns

In this study, we identified key mRNA, miRNA, and lncRNA molecules through high-throughput sequencing analysis and validated their expression patterns using qRT-PCR. The results revealed significant correlations in the expression changes of these molecules in the stroke model. Specifically, lncRNA expression exhibited an inverse relationship with miRNA expression, while showing a consistent trend with mRNA expression. This divergence in expression patterns is likely attributed to the regulation of mRNA expression by lncRNA via the "miRNA sponge" mechanism. Specifically, increased lncRNA expression can bind and inhibit miRNA activity, thereby weakening the suppressive effect of miRNA on its target mRNA, ultimately resulting in elevated mRNA expression levels.

2. Basis for Target Binding Site Predictions

To ensure the accuracy and reliability of the predicted target binding sites, we employed multiple established bioinformatics tools to stringently screen and analyze the binding sites of miRNAs with lncRNAs and mRNAs:

Prediction of miRNA and lncRNA: We utilized the miRDB database (http://www.mirdb.org/) to predict miRNAs associated with lncRNAs, applying screening criteria of a target score ≥ 60 and a conservation score > 0.5.

Prediction of miRNA and mRNA: To identify mRNAs complementary to miRNAs, we combined analyses from TargetScan (http://www.targetscan.org/) and miRanda (http://www.microrna.org/). The screening criteria included:

TargetScan context++ score ≤ -0.4

miRanda score > 140 and binding energy < -20 kcal/mol.

3. Subcellular Localization Distribution

The function of lncRNAs is closely linked to their subcellular localization. In this study, we analyzed the subcellular localization features of key lncRNAs using the lncLocator database. The results indicated that the identified lncRNAs predominantly localized to the cytoplasm. This localization suggests that these lncRNAs are likely to interact with miRNAs through the competitive endogenous RNA (ceRNA) mechanism, thus indirectly regulating mRNA expression. This information further supports our proposed regulatory network hypothesis.

4. Experimental Validation Support from Existing Literature

The key long non-coding RNAs (lncRNAs) and miRNAs identified through bioinformatics analysis in our study have received partial support from existing literature:

miR-370-3p: Studies have shown that miR-370-3p is downregulated following ischemia/reperfusion injury and has anti-angiogenic effects [1]. This finding aligns with our bioinformatics prediction regarding the regulatory role of miR-370-3p in ischemic injury.

miR-139-3p: Research by Yang et al. (2022) demonstrated that miR-139-3p is significantly upregulated after ischemic stroke. CircUSP36, a circular RNA, acts as a "sponge" for miR-139-3p, inhibiting its activity and thereby upregulating SMAD3 and Bcl2 expression, which reduces neuronal apoptosis [2]. These findings are consistent with our predicted miRNA-target gene regulatory network and further validate the accuracy of our bioinformatics analysis.

These studies provide important experimental support for our predicted regulatory network, highlighting the significant roles of key miRNAs in ischemic stroke while indirectly supporting the potential regulatory mechanisms involving key lncRNAs.

5. Characteristics of the Regulatory Network in Stroke as a Complex Disease

Ischemic stroke is a complex, multifactorial disease characterized by a cascade of responses to ischemia that is not a singular linear process. Instead, it involves interactions with various mechanisms and events [3-5]. Significant events such as hypoxia, oxidative stress, mitochondrial damage, inflammation, necrosis, and apoptosis are interconnected and may exert opposing biological effects, either providing neuroprotection or contributing to neurodamage. Ultimately, the impact of these events on disease prognosis and outcomes is influenced by a comprehensive balance of various factors concerning time, space, and intensity of action. Our experiments revealed significant differences in the expression patterns of lncRNAs in ischemic stroke. These lncRNAs participate in multiple mechanisms and events within the ischemic cascade, influencing relevant signaling pathways and major pathological mechanisms after stroke by regulating host gene expression at the transcriptional level . Given the dynamic complexity of stroke development, exploring the intricate network mechanisms involving multiple molecules, pathways, regulatory levels, and factors may provide a broader perspective for understanding this disease comprehensively. This systems-level approach aids in revealing key molecules and regulatory networks involved in the pathological processes of stroke, offering new theoretical frameworks and directions for future research and therapeutic strategies.

Additionally, our research group places great emphasis on functional and target validation experiments to further enhance the reliability of our bioinformatics predictions. Currently, we have initiated a series of functional validation experiments focusing on the identified key miRNAs and lncRNAs. However, due to the complexity of these experiments and time constraints, the studies remain incomplete, and some results are still being organized. In future research, we plan to further explore the functions of these miRNAs and lncRNAs, as well as their downstream regulatory mechanisms, to provide more comprehensive and robust experimental support for the predictions made in this study.

1.	Gu Y, Becker V, Zhao Y, Menger MD, Laschke MW. miR-370 inhibits the angiogenic activity of endothelial cells by targeting smoothened (SMO) and bone morphogenetic protein (BMP)-2. Faseb J. 2019;33(6):7213-24. http://doi.org/10.1096/fj.201802085RR

2.	Yang J, He W, Gu L, Long J, Zhu L, Zhang R, et al. CircUSP36 attenuates ischemic stroke injury through the miR-139-3p/SMAD3/Bcl2 signal axis. Clin Sci. 2022;136(12):953-71. http://doi.org/10.1042/CS20220157

3.Xing C., Arai K., Lo E. H., Hommel M. Pathophysiologic cascades in ischemic stroke. International Journal of Stroke: official journal of the International Stroke Society . 2012;7(5):378–385. doi: 10.1111/j.1747-4949.2012.00839.xIF: 6.3 Q1 .

4.Markus H. S. Stroke genetics. Human Molecular Genetics . 2011;20(R2):R124–R131. doi: 10.1093/hmg/ddr345IF: 3.1 Q2 .

5.Tang J. Y., Farooqi A. A., Ou-Yang F., et al. Oxidative stress-modulating drugs have preferential anticancer effects - involving the regulation of apoptosis, DNA damage, endoplasmic reticulum stress, autophagy, metabolism, and migration. Seminars in Cancer Biology . 2019;58 doi: 10.1016/j.semcancer.2018.08.010IF: 12.1 Q1 .

**Reviewer #2:**

Major Comments:

**2.1 Comment**: The authors investigated the roles of lncRNAs, miRNAs, and mRNAs in cerebral ischemia-reperfusion injury For this they employed the MCAO model in rats and analyzed differential gene expression profiles (lncRNAs, miRNAs, and mRNAs) using high-throughput sequencing. They determined the subcellular localization of lncRNAs and predicted miRNA targets using bioinformatics tools. The authors constructed an lncRNA-miRNA-mRNA regulatory network involved in microglial activation, apoptosis, angiogenesis, and inflammatory responses following stroke. The expression of key network components was validated using qPCR. This publication is well written, the coontrols are properly performed and the results are very interesting.

**Response**:We appreciate the positive evaluation of our work and are pleased that the reviewer recognizes the study design, results, and writing of our manuscript. Your affirmation serves as great encouragement for our team’s efforts.

**2.2** **Comment**:The specific number for Ethics Guidelines for Animal Experiments of the First Affiliated Hospital should be displayed in the core of the manuscript.

**Response:**Thank you for your valuable suggestion. We have updated the manuscript to include the specific approval number issued by the Institutional Animal Care and Use Committee (IACUC).

Modified text:

"All animal procedures were conducted following the guidelines established by the Institutional Animal Care and Use Committee (IACUC) of the First Affiliated Hospital of Shihezi University School of Medicine. The approval number is A2022-037-01. Animal care and use strictly followed the recommendations outlined in the Chinese National Regulations for Experimental Animal Management. This study report adheres to the ARRIVE guidelines."

**2.3 Comment:**Please justify the use of ACTB as an internal standard for lncRNAs and mRNAs, and U6 as an internal standard for miRNAs. Did you check that expression did not vary between conrol and stroke?

**Response:**Both lncRNA and mRNA originate from precursor RNA and undergo splicing in the nucleus, where introns are removed and exons are joined together to form mature RNA molecules. They can both undergo alternative splicing, resulting in the generation of different transcript variants[6]. Selecting appropriate reference genes is critical for ensuring the accuracy and reliability of gene expression studies. ACTB (β-actin) and U6 are widely recognized for their stability across different experimental conditions and tissue types. In molecular biology research, β-actin is extensively used as an endogenous control for the quantification of mRNA and lncRNA, while U6 serves as a normalization control for circulating miRNA analyses.

Recent studies on diabetic nephropathy (DN) have demonstrated that lncRNA MEG3 alleviates podocyte injury by inhibiting the Wnt/β-catenin signaling pathway, with β-actin utilized as an internal reference[7]. In addition, liquid biopsy studies have shown that the expression levels of lncRNA GAS5 and miR-625-3p, normalized to β-actin and U6 respectively, can serve as predictive biomarkers for the efficacy of neoadjuvant chemotherapy in patients with malignant pleural mesothelioma[8]. Moreover, GW4869 has been found to inhibit the production of lncRNA-ASLNCS5088-enriched exosomes by M2 macrophages, thereby reducing their regulatory impact on fibroblast activation, with expression levels normalized to β-actin for lncRNA and U6 for miRNA analyses[9]. Experimental validation has consistently demonstrated that both ACTB and U6 exhibit stable Ct values when comparing normal brain tissue to samples subjected to 2 hours of ischemia followed by 24 hours of reperfusion, further confirming their reliability as internal controls.

This study selects ACTB and U6 as reference genes based on their widespread use in the literature and experimental validation of their stability, in accordance with the scientific standards of qRT-PCR. Detailed experimental verification data and relevant literature citations will be included in the revised manuscript to further enhance the scientific rigor and persuasiveness of this study.

6.Melé M, Mattioli K, Mallard W, Shechner DM, Gerhardinger C, Rinn JL. Chromatin environment, transcriptional regulation, and splicing distinguish lincRNAs and mRNAs. Genome Research. 2017;27(1):27-37. doi:10.1101/gr.214205.116.

7.Wang M, Chen W, Geng Y, Xu C, Tao X, Zhang Y. Long Non-Coding RNA MEG3 Promotes Apoptosis of Vascular Cells and is Associated with Poor Prognosis in Ischemic Stroke. Journal of Atherosclerosis and Thrombosis. 2020;27(7):718-726. doi:10.5551/jat.50674.

8.Kresoja-Rakic J, Szpechcinski A, Kirschner MB, Ronner M, Minatel B, Martinez VD, et al. miR-625-3p and lncRNA GAS5 in Liquid Biopsies for Predicting the Outcome of Malignant Pleural Mesothelioma Patients Treated with Neo-Adjuvant Chemotherapy and Surgery. Non-Coding RNA. 2019;5(2): 41. doi:10.3390/ncrna5020041.

9.Chen J, Zhou R, Liang Y, Fu X, Wang D, Wang C. Blockade of lncRNA-ASLNCS5088-enriched exosome generation in M2 macrophages by GW4869 dampens the effect of M2 macrophages on orchestrating fibroblast activation. FASEB Journal. 2019;33(11):12200-12212. doi:10.1096/fj.201901610.

**2.4 Comment:**A publication has shown that miR-126 expression varies in CKD where alterations of cerebral circulation were linked with an increase in ischemic strokes PMID: 24389144. Did you check its expression in your experimental model please?

**Response**: We sincerely appreciate the reviewer's insightful comment regarding miR-126 and their attention to the important findings reported in PMID: 24389144 concerning the altered expression of miR-126 in CKD. This study highlights the downregulation of mature miR-126 in the cerebral microvasculature of CKD mice and its association with cerebrovascular alterations, which is of great significance for understanding the mechanisms underlying CKD-related cerebrovascular complications.

In our high-throughput sequencing data, we also identified a sequence related to miR-126, which we have termed "identified_miR-126" (sequence: aauaaagaacucagggguuggggauuuagcucaauggagaaugcuugccuagcaagcacaaggcccuggguucaguccccagcccccccaaaaaaagaaaagaaaaaa). It is crucial to emphasize that "identified_miR-126" is not the known mature miR-126-3p or miR-126-5p, but rather a novel sequence with a significantly greater length than the known pre-miR-126. In fact, "identified_miR-126" encompasses a portion of the pre-miR-126 sequence, but its overall length and remaining sequence differ from the known pre-miR-126.

Regarding the reviewer's question about the expression level of "identified_miR-126", we did not validate it in the current study. This is primarily due to the following consideration: at the initial stage of the project, we prioritized the study of known miRNAs with substantial literature support to facilitate bioinformatics analysis and literature interpretation. For newly identified sequences via sequencing, such as "identified_miR-126", we plan to conduct in-depth expression and functional validation in subsequent studies. Therefore, this study did not include experimental validation of the expression level of this sequence.

We acknowledge that this sequence shares partial overlap with the mouse miR-126 sequence. However, it is imperative to note that "identified_miR-126" is not the known miR-126, but rather a novel sequence identified based on our sequencing results. It may represent a novel precursor of pre-miR-126, a part of a longer transcript containing the pre-miR-126 sequence, or even a segment of pri-miR-126 that encompasses the pre-miR-126 region. Its precise nature and function require further investigation.

**Reviewer #3:**

**3.1.Comment:** The highly complex nature of bulk RNA sequencing will limit the exact cellular origin of the investigated RNA molecules.

**Response :**

Thank you for reviewing our manuscript and for your valuable feedback. Regarding your concerns about the high complexity of RNA sequencing and its limitations in determining the precise cellular sources of RNA molecules, we appreciate your insights. Below is our response, specifically addressing the use of rat cortical tissue samples:

1.Tissue Collection and Processing: During the collection of rat cortical tissue, we minimized contamination from other brain regions. We employed cryosectioning techniques to ensure tissue integrity and reduce cellular damage during processing.

2.RNA Extraction and Sequencing: We utilized an efficient RNA extraction method to ensure high quality and purity of RNA from the cortical tissue. During RNA sequencing, we implemented high-throughput sequencing technologies and conducted rigorous quality control and filtering of the data.

3.Data Analysis and Calibration: In the data analysis phase, we employed various bioinformatics tools and databases to confirm the accuracy of our sequencing results. For instance, we performed alignment and annotation of the sequencing data, using known genomic information to calibrate and validate our findings.

4.Biological Validation: We validated the biological relevance of our sequencing results by comparing them with existing gene expression databases, confirming that the majority of highly expressed genes align with the biological functions of cortical tissue. Additionally, we plan to conduct supplementary experiments, such as quantitative PCR (qPCR), to verify the expression levels of specific genes.

**3.2.Comment:**The ceRNA hypothesis has its limitations. The authors need to discuss the merits of the findings in the light of the issues raised by eg. Yhomson and Dinger NRG 2016. (Thomson, D., Dinger, M. Endogenous microRNA sponges: evidence and controversy. Nat Rev Genet 17, 272–283 (2016).doi: 10.1038/nrg.2016.20)

**Response :**Thank We would like to express our sincere gratitude for your thorough review and valuable feedback on our research. We acknowledge the limitations of the ceRNA hypothesis that you highlighted, particularly the concerns raised by Thomson and Dinger (2016). In our study, we have recognized and addressed these limitations, which we discuss in detail below:

1. Limitations of Experimental Validation

We understand that reliance on exogenous overexpression systems may result in a lack of physiological relevance. To overcome this issue, we validated the endogenous expression patterns of key molecules (lncRNA, miRNA, and mRNA) in a stroke model using high-throughput sequencing and qRT-PCR. This approach effectively mitigated biases that could arise from overexpression and enhanced the physiological relevance of our data.

Regarding the limitations of AGO immunoprecipitation techniques, we analyzed the subcellular localization of key lncRNAs in our study. The results indicated that these lncRNAs primarily localize to the cytoplasm, which is consistent with their role in regulating mRNA expression through the ceRNA mechanism, thus providing indirect support for our regulatory network hypothesis.

Additionally, we conducted an in-depth exploration of the distinction between the coding functions of mRNA and their non-coding functions as ceRNAs. We employed bioinformatics tools to rigorously screen miRNA binding sites to ensure the specificity of our predictions, supported by literature references (such as the functional validations of miR-370-3p and miR-139-3p), which further enhanced the reliability of our results.

2. Limitations of Bioinformatics Modeling

In terms of bioinformatics modeling, we recognize the shortcomings of miRNA target prediction algorithms. Most existing algorithms, such as TargetScan and miRanda, primarily focus on the 3′UTR region of mRNA, potentially overlooking other relevant binding sites. Therefore, we utilized a combination of multiple algorithms in our study and performed stringent screening and cross-validation of miRNA binding sites with lncRNAs and mRNAs to ensure the high credibility of our predictions.

3. Research Highlights

Ischemic stroke is a complex, multifactorial disease characterized by a non-linear cascade of responses to ischemia, involving interactions among various mechanisms and events. Key events such as hypoxia, oxidative stress, mitochondrial damage, inflammation, necrosis, and apoptosis are interconnected and may exert opposing biological effects, providing neuroprotection or contributing to neuronal damage. The ultimate impact of these events is determined by a comprehensive balance of multiple factors, including timing, spatial dynamics, and intensity of action.

Our study reveals significant differences in the expression patterns of long non-coding RNAs (lncRNAs) in ischemic stroke, highlighting their potential roles in the disease. These lncRNAs may regulate host gene expression at the transcriptional level, thereby influencing relevant signaling pathways and major pathological mechanisms, reflecting the complexity of the ischemic cascade. By generating rich expression data through high-throughput sequencing and employing various bioinformatics tools for cross-analysis, we constructed a ceRNA network.

Moreover, the predicted roles of lncRNAs and miRNAs in inflammatory responses, neuroprotection, and tissue repair may provide new insights into the pathological mechanisms of ischemic stroke and offer potential targets for developing novel therapeutic strategies. The constructed ceRNA regulatory network illustrates the complex interactions among these molecules, emphasizing their cooperative roles in stroke. This systems biology approach aids in further elucidating the roles of these molecules in disease progression and establishes a foundation for future research. By modulating specific lncRNAs or miRNAs, we may influence neuroprotection and recovery processes following ischemic stroke, thereby providing new strategies to improve patient outcomes.

**3.3 Method section**

**3.3.1 Comment**:2.3 TTC staining How are the images acquired?

**Response**:Thank you for pointing this out. TTC staining relies on the activity of dehydrogenases within the mitochondria of viable cells. These enzymes reduce colorless TTC (2,3,5-Triphenyltetrazolium Chloride) to an insoluble red formazan. Due to the reduction or loss of dehydrogenase activity in ischemic tissue, it fails to reduce TTC. Consequently, normal tissue stains red, whereas ischemic tissue appears white. We have added detailed information about image acquisition methods.

Modified text:

The rats were sacrificed, and the whole rat brains were collected and stored at -20°C for 30 minutes. The brains were then cut into 2 mm thick coronal slices. The brain slices were subsequently stained with 2% TTC at 37°C in the dark for 30 minutes, with flipping every 8 minutes to ensure uniform exposure to the TTC staining solution. Images were acquired using a digital camera (Canon EOS 800D) under consistent lighting conditions. We used ImageJ to measure the infarct area and calculated the infarct volume percentage as the infarct volume/total volume of the contralateral hemisphere × 100%.

**3.3.2 Comment**:2.4 Transcriptome sequencing

Very limited information on the sequencing. The authors need to provide more details on the kits used and bioinformatic tools used for processing the reads and alignments.Does the mRNA and lncRNA counts come from the same RNA sequencing libraries?What databases of annotations are used?

**Response**:2.4 Transcriptome sequencing:

We have added detailed information about library construction and sequencing protocols. Two separate libraries were constructed: one for miRNA and another for mRNA/lncRNA sequencing. The detailed bioinformatics pipeline has been added to Section 2.4 of the Methods.The library construction process is depicted in Supplementary Figure 1.

Supplementary Figure 1.Library construction and sequencing of lncRNA, miRNA, and mRNA.

**3.3.3 Comment**:2.6 MiRNA and mRNA identification

How are the microRNA sequenced? What reference genome? How are novel microRNAs identified? The part with identification of mRNAs in this part is very unclear to me. I don’t understand the connection to the microRNA sequencing and quantication. Please split this part up.

**Response**:2.6 MiRNA and mRNA identification

Thank you for your comments and suggestions. We have reorganized the identification sections for miRNA and mRNA to clearly distinguish between the two analyses. Information regarding the sequencing process for microRNA has been added to Methods Section 2.4, which includes details on the high-throughput sequencing method, quality control steps, and alignment procedures. The reference genome used is Rattus norvegicus Rnor_5.0, which serves as the basis for aligning miRNA sequences and annotating their locations; this information has been included in Methods Section 2.6. Additionally, details on how to identify novel miRNAs have also been incorporated into Methods Section 2.6. We have separated the identification sections for miRNAs and lncRNAs, with miRNA identification now included in Section 2.6 and mRNA identification in Section 2.7, to enhance the clarity and logical flow of the analysis.

**3.3.4 Comment**:2.7 Screening of differentially expressed lncRNAs, miRNAs and mRNAs

I don’t like the word screening. Please consider changes it to identification of differentially expressed *RNA.

**Response**:Thank you for your suggestion. We agree that "identification" is more appropriate than "screening" in this context. We have changed the section heading from "Screening of differentially expressed lncRNAs, miRNAs and mRNAs" to "Identification of differentially expressed lncRNAs, miRNAs and mRNAs" to better reflect the analytical nature of this process.Due to the separation of the identification sections for miRNAs and lncRNAs, the numbering of subsequent sections has been shifted by one position.

Modified text:

"2.8 Identification of differentially expressed lncRNAs, miRNAs and mRNAs"

**3.3.5 Comment**:2.10 CeRNA network analysis

Please elaborate on this part. How are the interactions scored from the different target prediction algorithms?

**Response**:Thank you for your suggestion. We have detailed the scoring criteria used in different prediction algorithms, and this information is now included in Methods Section 2.11.

1. lncRNA-miRNA Interaction Prediction

The miRDB database (http://www.mirdb.org/) was used to predict miRNAs corresponding to lncRNAs with a minimum target score of 60 and conservation scores > 0.5[10].

2. miRNA-mRNA Interaction Screening

TargetScan (http://www.targetscan.org/) and miRanda (http://www.microrna.org/) were used to screen for mRNAs that are complementary to the miRNAs, with TargetScan context++ scores ≤ -0.4 [11]and miRanda scores > 140 (energy < -20 kcal/mol)[12]. Only targets predicted by both algorithms were retained. The ceRNA network of lncRNAs/miRNAs/mRNAs was visualized using Cytoscape (version 3.9.1).

10. Wong N, Wang X. miRDB: an online resource for microRNA target prediction and functional annotations. Nucleic Acids Res. 2015 Jan;43(Database issue):D146-52. doi: 10.1093/nar/gku1104IF: 16.6 Q1 . Epub 2014 Nov 5. PMID: 25378301; PMCID: PMC4383922.
11. Lewis BP, Burge CB, Bartel DP. Conserved seed pairing, often flanked by adenosines, indicates that thousands of human genes are microRNA targets. Cell. 2005 Jan 14;120(1):15-20. doi: 10.1016/j.cell.2004.12.035IF: 45.5 Q1 . PMID: 15652477.
12. Enright AJ, John B, Gaul U, Tuschl T, Sander C, Marks DS. MicroRNA targets in Drosophila. Genome Biol. 2003;5(1):R1. doi: 10.1186/gb-2003-5-1-r1IF: 10.1 Q1 . Epub 2003 Dec 12. PMID: 14709173; PMCID: PMC395733.

**3.3.6 Comment**:2.12 Validation of the RNA-seq results using quantitative PCR (qPCR)

Im not sure I understand the first part with the nested PCR? How much RNA is used as input for the cDNA reactions? Are the cDNA diluted?

**Response**:2.12 Validation of RNA-seq results using qPCR

Nested PCR is an optimized polymerase chain reaction technique designed to enhance both the specificity and sensitivity of DNA amplification. The procedure consists of two sequential stages: first, a pair of external primers located at both ends of the target sequence is used for an initial amplification, generating a larger DNA fragment. Subsequently, the product from the first PCR is used as the template for a second amplification with a pair of internal, nested primers situated within the initial primer regions. Because the nested primers can only anneal to sequences within the specific region amplified during the first PCR, the second amplification primarily targets the desired region, thereby significantly reducing the formation of non-specific products[13]. This dual-layer primer approach is particularly advantageous for samples with low target sequence concentrations or those containing a substantial amount of non-specific DNA, as it markedly improves both the specificity and sensitivity of the amplification and subsequent detection. Consequently, nested PCR is widely valuable in gene detection and validation processes that require high precision and sensitivity.

Thank you for requesting clarification about the nested PCR protocol and cDNA preparation. We have revised this section to provide more detailed information about RNA input amounts and cDNA dilution steps.We have incorporated the revised text into Section 2.13 of the Methods, as suggested by the reviewers.

13.Green MR, Sambrook J. Nested Polymerase Chain Reaction (PCR). Cold Spring Harb Protoc. 2019 Feb 1;2019(2). doi: 10.1101/pdb.prot095182. PMID: 30710024.

**3.4.Results**

**3.4.1 Comment**:Line 306: multiple test corrected p-value or p-value?

Response:Thank you for raising this important question. We confirm that adjusted p-values (FDR corrected using the Benjamini-Hochberg method) were used for the differential expression analysis. We have clarified this point in the manuscript and included the revised information in the Results section, specifically in Section 3.3: Identification of differentially expressed miRNAs and mRNAs at line 370 in the Revised Manuscript with Track Changes.

**3.4.2 Comment**:Line 307: DEmRNA?

**Response:**Thank you for pointing this out. We apologize for the typo in line 307. The correct term should be "DEmiRNA" (differentially expressed microRNA), not "DEmRNA." This has been corrected for clarity and accuracy and is reflected at line 371 in the Revised Manuscript with Track Changes.

**3.4.3 Comment**:Line 327: It is unclear what is meant here? lncRNA and microRNAs in general or specifically for microRNAs with targets in lncRNA? This needs to be supported by an analysis. Are there any enrichment of positive or negative correlations among lncRNA with target sites for microRNAs?

**Response:**We sincerely thank the reviewer for their valuable comments on the ceRNA theory described in our manuscript. Below are our specific responses:

Our discussion regarding the relationship between lncRNAs and miRNAs is primarily based on the core mechanism of the ceRNA theory, focusing specifically on lncRNAs containing miRNA response elements (MREs) that competitively bind miRNAs, rather than addressing a generalized interaction between all lncRNAs and miRNAs. Through binding with miRNAs via hydrogen bonds, lncRNAs act as "sponges," reducing the inhibitory effects of miRNAs on their target RNAs (e.g., mRNAs). When lncRNA levels decrease, fewer MREs are available to bind miRNAs, resulting in more free miRNAs that can interact with other target RNAs, thereby enhancing their repression of mRNA expression[14-15]. This mechanism highlights that the regulatory relationship between miRNAs and lncRNAs is specific to lncRNAs with MREs and does not apply to all lncRNAs.

In terms of experimental validation, we identified key mRNAs, miRNAs, and lncRNAs through high-throughput sequencing and further confirmed their expression patterns using qRT-PCR. In the stroke model, lncRNA expression was inversely correlated with miRNA levels, while showing a positive correlation with mRNA expression. These findings support the hypothesis that lncRNAs indirectly promote mRNA expression through the "miRNA sponge" mechanism. To further validate the interactions between lncRNAs, miRNAs, and mRNAs, we utilized multiple bioinformatics tools with stringent criteria. For instance, MRE sites in lncRNAs were predicted using the miRDB database (target score ≥ 60 and conservation score > 0.5), while miRNA-mRNA interactions were identified using combined analyses from TargetScan (context++ score ≤ -0.4) and miRanda (score > 140 and binding energy < -20 kcal/mol). These analyses provided a robust foundation for investigating the ceRNA mechanism by which lncRNAs regulate mRNA via miRNA sequestration. Additionally, using the lncLocator database, we analyzed the subcellular localization of key lncRNAs and found that they predominantly localize to the cytoplasm, further supporting their potential to interact with miRNAs through the ceRNA mechanism and indirectly regulate mRNA expression.

Stroke is a complex, multifactorial disease involving pathological processes such as hypoxia, oxidative stress, inflammation, mitochondrial dysfunction, and apoptosis. We observed that lncRNAs play critical roles in regulating host gene expression, participating in multiple pathological mechanisms and signaling pathways post-stroke. By competitively binding miRNAs, lncRNAs integrate various pathways into a complex regulatory network. These dynamic regulatory features offer new insights into the molecular mechanisms underlying stroke pathology and provide a novel perspective for identifying key molecules and networks involved in the disease process.

Modified Placement: Section 3.4 Construction of the ceRNA Network in Stroke

14.	Salmena L, Poliseno L, Tay Y, Kats L, Pandolfi PP. A ceRNA hypothesis: the Rosetta Stone of a hidden RNA language? Cell. 2011;146(3):353-8. http://doi.org/10.1016/j.cell.2011.07.014

15.	Tay Y, Rinn J, Pandolfi PP. The multilayered complexity of ceRNA crosstalk and competition. Nature. 2014;505(7483):344-52. http://doi.org/10.1038/nature12986

**3.3.4 Comment**:Line 340: How did you confirm the 634 genes as differentially expressed target mRNAs?

**Response:**

Thank you for this insightful question. Here is how we identified the 634 differentially expressed target mRNAs:

High-throughput RNA sequencing initially identified 2,608 differentially expressed mRNAs (DEmRNAs). To identify target mRNAs, we performed miRNA target prediction using TargetScan and miRanda. The predicted mRNA targets of the differentially expressed miRNAs (DEmiRNAs) were then compared with the 2,608 DEmRNAs. The intersection of these two datasets represents the 634 differentially expressed target mRNAs, which are both differentially expressed and predicted to be regulated by the DEmiRNAs, thereby confirming their status as potential target mRNAs.

**3.4.5 Comment**:Line 417: How does MSTRG.151823.1 modulate the expression of the genes?

**Response:**

According to our predictions, MSTRG.151823.1 regulates the expression of multiple genes by modulating the expression of miR-370-3p. Specifically, MSTRG.151823.1 downregulates miR-370-3p, which targets several genes, including Bag3, Serpine1, Il6r, Flna, and Ptgs2.

Our experimental results show that MSTRG.151823.1 is upregulated, while miR-370-3p is downregulated. Concurrently, we observed an increase in the expression of Bag3, Serpine1, and Il6r. Based on literature reviews, we found that Bag3 and Serpine1 may have anti-apoptotic effects, while Il6r may be involved in promoting apoptosis. Therefore, MSTRG.151823.1 may downregulate miR-370-3p, leading to the upregulation of Bag3 and Serpine1, which in turn inhibits apoptosis, while also upregulating Il6r, which promotes apoptosis.

In terms of angiogenesis, MSTRG.151823.1 may downregulate miR-370-3p, resulting in the upregulation of Flna, which promotes angiogenesis. However, MSTRG.151823.1 may also downregulate Ptgs2 and Serpine1, which in turn inhibits angiogenesis. This complex regulatory mechanism suggests that MSTRG.151823.1 plays a crucial role in regulating apoptosis and angiogenesis.

A single lncRNA can simultaneously regulate the expression of multiple genes, which may have distinct functions and mechanisms, leading to complex biological effects. This phenomenon is common in gene regulatory networks, reflecting the complexity and diversity of gene regulation. Our results further support this notion, indicating that MSTRG.151823.1's regulatory mechanism involves multiple genes and signaling pathways. Further research will help us better understand its regulatory mechanism and biological function, revealing its role in apoptosis and angiogenesis.

**3.4.6 Comment**:Line 278: It is unclear what is done here? This needs to be descibed in details in the methods section.Line 318: This strategy is unclear to me and needs to be described in details in the methods section?

Line 341: this strategy is unclear to me and needs to be described in details in the methods section.

**Response :**Thank you for your comments and suggestions. We agree that providing additional details regarding the strategies and processes used in our study is necessary for clarity. To address your concerns, we have now included a detailed description of the methods in the revised manuscript. Additionally, to further clarify our workflow, we have created a flowchart (Supplementary Figure 2) to visually outline the key steps and strategies used in our analysis.

Supplementary Figure 2.The lncRNA-miRNA-mRNA Regulatory Network

**3.4.7.Comment**:The supplementary material lacks the count data from the microRNA expression analsysis.

Thank you for your valuable feedback regarding the supplementary material. We sincerely apologize for the oversight in not including the count data from the microRNA expression analysis. We have now uploaded the relevant count data as Supplementary Table S19.

**3.4.8.Comment**:Some of the primers in supplementary materials “S1 Table. Primer sequence” seems wrong. (miR-351-5p RT and miR-674-5p RT).

**Response**:Thank you for reviewing our manuscript and for your valuable feedback. Regarding your concern about potential errors in the primer sequences listed in Table S1, we take your feedback very seriously and would like to provide our response:

We carefully checked the primer sequences in Table S1, particularly the designs for the miR-351-5p RT and miR-674-5p RT primers. After thorough verification, we discovered that there was indeed a copy-paste error. We have corrected the relevant primer sequences in Table S1 to ensure their accuracy.

**Reviewer #4:**

**4.1** **Comment**:lncRNAs and protein-coding genes could be analyzed using one RNA-seq library, while library for small noncoding RNAs is usually constructed separately. Authors analyzed differentially expressed protein coding-genes and miRNAs together using the same library? Moreover, only one Transcriptome sequencing protocol in Materials and Methods section, so it looks like authors established one library for all purposes.

**Response**:Thank you for your careful observation. We have added detailed information about library construction and sequencing protocols. Two separate libraries were constructed: one for miRNA and another for mRNA/lncRNA sequencing. The detailed bioinformatics pipeline has been added to Section 2.4 of the Methods.The library construction process is depicted in Supplementary Figure 1.

**4.2** Comment:Too many typos, especially page 15 “we identified 2,145 miRNAs, 19.91% of which were known miRNAs. By applying thresholds of p < 0.05 and |log2FC| > 1, we identified 651 DEmRNAs between the MCAO and sham groups, with 464 upregulated and 187 downregulated miRNAs (S9 Table) (Figure 4A, B). Additionally, using stricter criteria of |log2FC| > 2 and FDR < 0.01, we identified 2,608 DE miRNAs, including 1,133 upregulated and 1,475 downregulated mRNAs”, it is confusing.

**Response**:Thank you for pointing out these inconsistencies. We apologize for the confusing description and have revised the text to ensure accuracy and clarity:
